# Feasibility of aortic aneurysm sac embolization using a novel shape memory polymer embolic device

**DOI:** 10.1186/s41747-023-00328-x

**Published:** 2023-04-03

**Authors:** Alexander Massmann, Peter Fries, Roushanak Shayesteh-Kheslat, Arno Buecker, Patrick Berg, Felix Frenzel

**Affiliations:** 1 Radiology and Nuclear Medicine, Bosch Health Campus, Robert-Bosch-Krankenhaus, Auerbachstraße 110, 70376 Stuttgart, Germany; 2 Diagnostic and Interventional Radiology, Saarland University Medical Center, Homburg/Saar, Germany; 3 Clinic for General, Visceral, Vascular and Pediatric Surgery, Saarland University Medical Center, Homburg/Saar, Germany; 4 Clinic for Vascular Surgery, Katholisches Karl-Leisner-Klinikum, Kevelaer, Germany

**Keywords:** Aortic aneurysm, Endoleak, Endovascular procedures, Polymers, Retrospective studies

## Abstract

**Background:**

We investigated the feasibility of aneurysm sac embolization using a novel self-expanding porous shape memory polymer (SMP) device during endovascular aortic abdominal or thoracic aneurysm repair (EVAR).

**Methods:**

Retrospective analysis of consecutive patients treated at 2 centers in Germany. Patients were treated from January 2019 to July 2021 with follow-up at 7 days and 3, 6, and 12 months. Aneurysm sacs were implanted with SMP devices immediately following endograft placement during the same procedure. Primary endpoint was technically successful SMP-device deployment into the aneurysm sac outside the endograft. Secondary endpoints were changes in aneurysm volume and associated complications (*e.g.*, endoleaks).

**Results:**

We included 18 patients (16 males), aged 72 ± 9 years, achieving 100% technical success. Mean preprocedure aortic aneurysm sac volume was 195 ± 117 mL with a perfused aneurysm volume of 97 ± 60 mL. A mean of 24 ± 12 SMP devices per patient were used (range 5–45, corresponding to 6.25–56.25 mL expanded embolic material volume). All evaluable patients exhibited sac regression except 2 patients yet to reach 3-month follow-up. At mean 11 ± 7 months (range 3–24), change in aneurysm volume from baseline was -30 ± 21 mL (*p* < 0.001). In 8 patients, aneurysm regression was observed despite type 2 endoleaks in 6 and type 1A endoleaks in 2, none of them requiring further intervention to date. No morbidity or mortality related to this treatment occurred.

**Conclusions:**

SMP devices for aortic aneurysm sac embolization during endovascular repair appear feasible and safe in this small case series. Prospective studies are needed.

**Key points:**

• Shape memory polymer is a novel, self-expanding, porous, and radiolucent embolic device material.

• Aortic aneurysm sacs were treated with polymer devices immediately following endograft placement.

• Aortic aneurysm sac regression was observed in all patients with over 3-month follow-up.

• Aortic aneurysm sac regression was observed even in the presence of endoleaks.

**Supplementary Information:**

The online version contains supplementary material available at 10.1186/s41747-023-00328-x.

## Background

Abdominal aortic aneurysm (AAA) sac failure to regress after endovascular aneurysm repair (EVAR) has been linked to increased long-term mortality [1, 2]. It is widely acknowledged that endoleaks contribute to sac expansion or failure to regress [3]. However, an absence of endoleak is not necessarily predictive of sac stability or regression [1, 2], and AAA sac management during and after EVAR remains a subject of substantial investigation.

Endoleak management, particularly for type 2, falls into two types, active and reactive, where the latter is currently more common practice. Reactive endoleak management options include a variety of techniques to reestablish endograft seals and/or aneurysm sac and/or branch vessel embolization techniques. Post-EVAR reactive sac/vessel embolization methods include transarterial and translumbar (direct injection) approaches. Embolic devices commonly used to embolize AAA sacs to treat endoleaks include peripheral vascular embolization coils, cyanoacrylate or fibrin glue, gelfoam [4–6], and Onyx [7–12]. Active AAA sac management options, *i.e.*, at the same time as EVAR, include the use of the Nellix system in the endovascular aneurysm sealing procedure [13–16], intraprocedural branch vessel embolization [17–22], and intraprocedural sac embolization with coils, thrombin, fibrin glue, and/or gelfoam [23–33].

Ultra-low-density polyurethane shape memory polymer is a novel embolic material now available as vascular plugs [34–36]. Shape memory polymer devices (IMPEDE-FX Embolization Plug, Shape Memory Medical, Santa Clara, CA, USA) are supplied in a stable crimped form for catheter delivery, and the devices self-expand to a specific volume upon exposure to blood, as they “remember” the manufactured shape. Porcine model studies characterized an acute inflammatory response to the polymer and the formation of a thrombus throughout the expanded porous structure. Progressive healing over 90 days resulted in noninflammatory replacement of shape memory polymer with cellular, collagenous connective tissue and stable vessel occlusion without microscopic indication of a sustained chronic-active inflammatory response [37]. Separately, a porcine sidewall aneurysm model experiment showed distinctly greater regression in aneurysm sacs treated with shape memory polymer than when treated with embolic coils 180-day post-implantation [38].

The purpose of this study was to determine the feasibility of using shape memory polymer vascular plugs for the embolization of aortic aneurysm sacs, to report on the safety of implanting the devices, and to report on preliminary efficacy in terms of changes in sac size and the rate of complications commonly associated with EVAR.

## Methods

### Study design

Retrospective analysis of consecutive patients treated with aortic aneurysm sac embolization at the same time as EVAR at 2 vascular centers in Germany, at an academic tertiary-level university and a teaching hospital. Patients were treated between January 2019 and July 2021. All patients gave written informed consent for the intervention, and local ethics committee approval was obtained for retrospective analysis. Anonymized data were independently reviewed by 2 experienced radiologists (A.M., F.F.).

Patients were candidates for elective EVAR treatment with perfused aneurysmal lumens large enough after EVAR to manipulate the delivery sheath and accommodate the delivery of embolization devices (per the device description and delivery procedure outlined below) and the absence of relevant calcifications of iliac access vessels to enable a parallel wire approach to the aneurysmal lumen using large transfemoral access sheaths. Furthermore, the large aneurysm sacs had a non-thrombosed aneurysm lumen/a low thrombus burden. Based on the experience of the interventionalists, these were considered to potentially benefit from sac embolization.

The primary endpoint was technical success, defined as the deployment of shape memory polymer devices in an aortic aneurysm sac outside of an aortic endograft in the same procedure. Secondary endpoints were changes in aneurysm sac volume, the rate of endoleaks, and procedure-related morbidity and mortality. Procedure time and fluoroscopy time were recorded, and inflammation markers (body temperature, blood sedimentation rate, leucocyte count) were also evaluated at 7 days before the procedure and in line with the follow-up imaging schedule. An additional timer was used to record the additional time used for sac embolization in each case.

### Imaging data

Preprocedural contrast-enhanced computed tomography angiography (CTA) image analysis and volumetry were performed in all patients on a dedicated workstation (Siemens Healthineers Syngo.Via, Erlangen, Germany). Follow-up imaging included combined contrast-enhanced ultrasound (CEUS) and multiphase computed tomography (CT) (non-contrast CT, arterial and venous phase, or perfusion CTA) at 7 days and 3, 6, and 12 months after the procedure, based on institutional standard of care. Critical nephropathy was not present in the treated patients. Annual follow-up through 5 years postprocedure is ongoing.

**Fig. 1:**
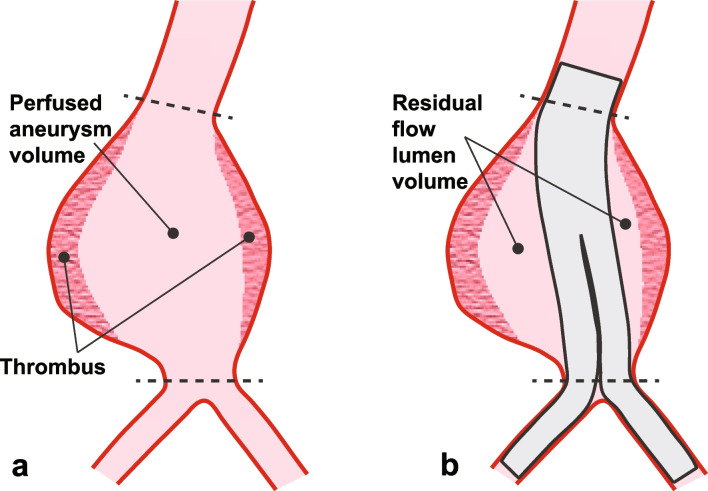
Schematic of the volumes associated with preparation for sac embolization. **a** Three-dimensional aneurysm volume was measured between positions of non-aneurysmal aorta diameter proximal and distal to the aneurysm (dotted lines, with applicable adjustments for individual anatomy). Perfused aneurysm volume was the aneurysm volume exclusive of preexisting thrombus. **b** The residual flow lumen volume was calculated by subtraction of the endograft volume (estimated from endograft dimensions in each product’s instructions for use) from the perfused aneurysm volume

All reported aneurysm measurements were based on CTA imaging analysis. Three-dimensional aneurysm volume was measured between positions of non-aneurysmal aorta diameter proximal and distal to the aneurysm. Perfused aneurysm volume was the aneurysm volume exclusive of preexisting thrombus. The residual flow lumen volume was calculated by subtraction of the endograft volume (estimated from endograft dimensions in each product’s instructions for use) from the perfused aneurysm volume (Fig. 1).

### Devices

**Fig. 2:**
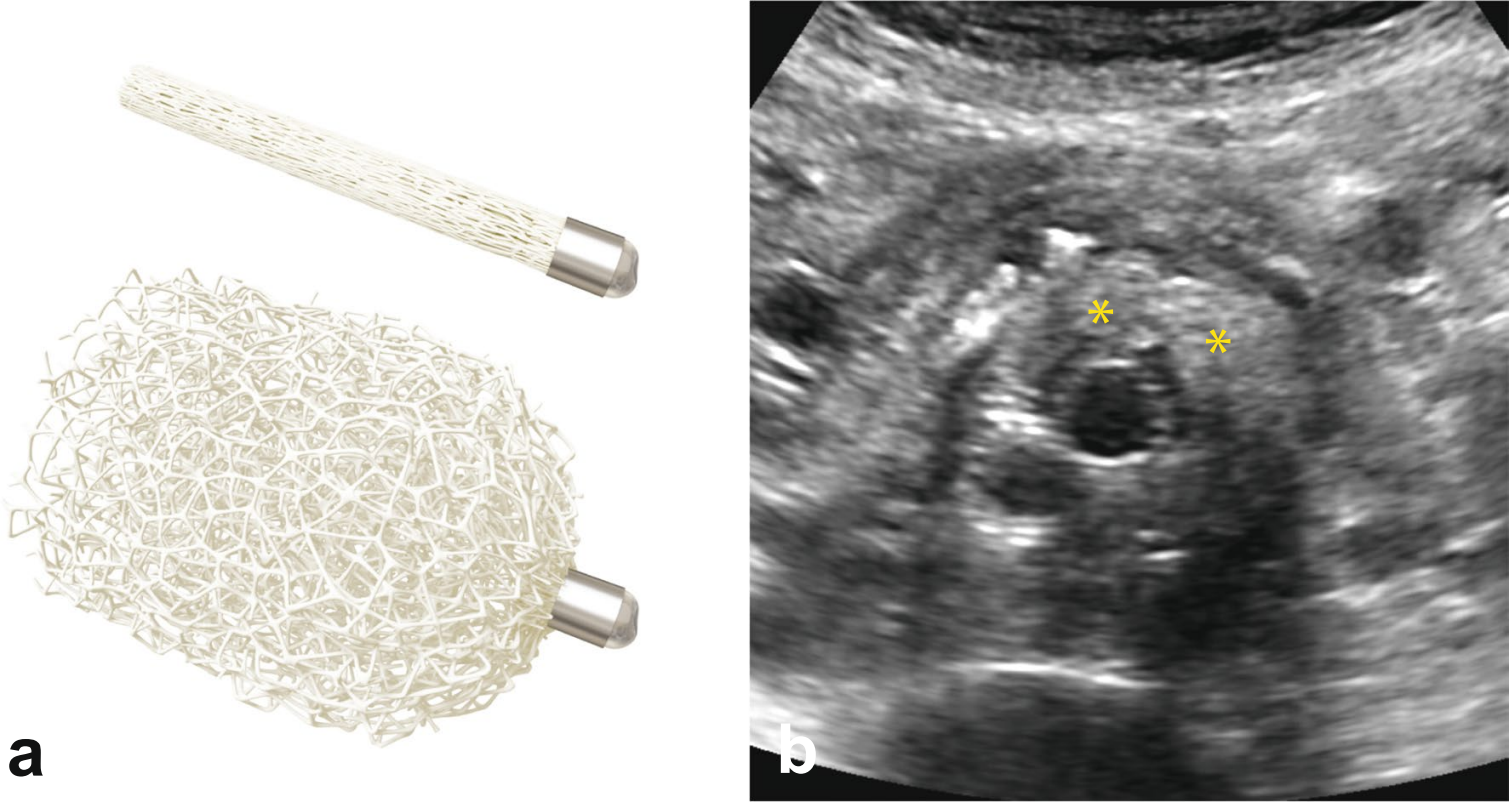
**a** Illustration of the implanted shape memory polymer device in its crimped (above) and (below) expanded form. **b** The appearance of shape memory polymer devices under follow-up ultrasound (yellow asterisks indicate location of some polymer). A video of shape memory polymer device deployment illustrating the appearance under fluoroscopy is shown in the supplementary material

Aortic endografts were selected based on case-specific needs. The shape memory polymer devices were 12-mm diameter IMPEDE-FX Embolization Plugs, as shown in Fig. 2. The shape memory polymer is crimped for catheter delivery and self-expands to a porous structure upon contact with blood. Each 12-mm diameter IMPEDE-FX device has a volume of ~ 1.25 mL when fully expanded. The shape memory polymer is radiolucent, and the devices are visualized under fluoroscopy via a small proximal radiopaque marker (Fig. 2). These devices were used both for sac embolization and the occlusion of aortic side branch ostia. The majority of cases were performed using individual IMPEDE-FX Embolization Plugs; three of the cases were performed with the IMPEDE-FX RapidFill device after it became available, in which 5 IMPEDE-FX Embolization Plugs are loaded in an introducer and implanted at the same time.

### Sac embolization procedure

**Fig. 3:**
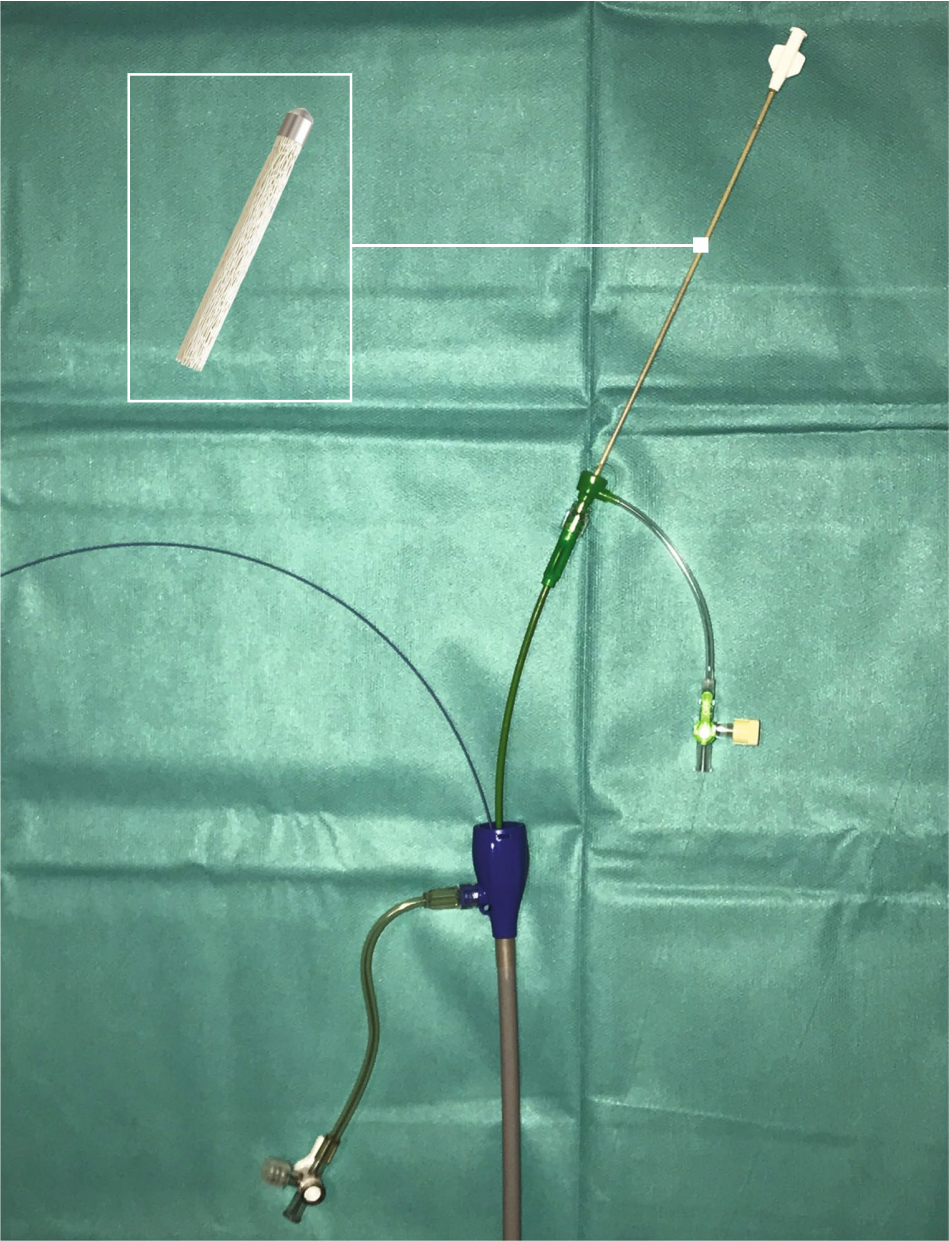
Large transfemoral access sheath for aortic endograft and a parallel wire with 6-Fr sheath for shape memory polymer device deployment into the aneurysm sac behind the endograft. Inset: illustration of the crimped form of the shape memory polymer device, which is supplied in its introducer

Procedures were performed by experienced interventional radiologists only. An additional timer was used to record the additional time used for sac embolization in each case. A transfemoral parallel wire technique was used to maintain access to the aneurysm sac after deployment of aortic endografts. For this purpose, an upsized introducer sheath was used (GORE DrySeal Flex, Flagstaff, Arizona, USA; or Medtronic Sentrant, Minneapolis, MN, USA), with a 2-Fr larger inner diameter than was indicated for the EVAR procedure. This allowed for simultaneous insertion of an 0.89-mm/0.035″ aneurysm access wire (Terumo Glidewire Advantage, Tokyo, Japan) and endograft delivery catheter via a single transfemoral percutaneous access sheath (Fig. 3). After withdrawal of the endograft delivery catheter, the parallel wire (in the aneurysm sac only) was used to advance a flexible, angulated, 6-Fr sheath with a smooth transition (Terumo Destination Guiding Sheath, Tokyo, Japan) adjacent to the deployed endografts into the aneurysm sac. The instructions for use recommend a minimum 0.070″ inner diameter delivery catheter for the 12-mm IMPEDE-FX Embolization Plugs, and the chosen 6-Fr sheath met the criterion.

Devices were delivered with an intention to fill the sac with embolic material as much as possible. Devices were distributed throughout the sac by initial arc-shaped insertion of the flexible sheath across the aneurysm from one to the other side and stepwise retraction during the deployment process. In case of device clustering, a standard 0.89 mm/0.035″ wire was used to distribute the devices throughout the non-thrombosed aneurysm sac. The supplementary material shows a video of representative device deployment. In cases with additional targeted occlusion of aortic side branch ostia (inferior mesenteric, internal iliac, or lumbar arteries), a small number of devices were delivered right at the ostium of the side branch to encourage thrombosis, where the ostia locations were identified in preprocedural CTA and catheter angiography prior to aortic endograft deployment. This is distinctly different from branch vessel embolization remote from the sac via vessel cannulation. The decision criterion for ostia embolization was the presence of a prominent aortic side branch diameter > 3 mm, which is known to be prone to type 2 endoleak. In the single case including placement of the devices at the ostium of the internal iliac artery, devices were placed behind the endograft limb as the delivery catheter was withdrawn. Following deployment of the shape memory polymer devices, the endograft placement was completed with ballooning of the limbs, completion angiography, and vessel closure.

Periprocedural standard medication included 5000 international units of heparin and a prophylactic single-shot antibiosis. Therapeutic heparinization (body-weight-adapted subcutaneous injection of low-molecular-weight heparin) was continued for 48 h, and acetylsalicylic acid 100 mg/day is prescribed for life.

### Data analysis

Percent change in follow-up aneurysm volumes was based on preprocedure baseline data. The mean percent change was calculated as the mean of individual percent changes in volume from baseline to follow-up. Continuous data are reported as the mean ± standard deviation and range (minimum − maximum). Categorical data are reported as number (percent of total). Continuous sac volume data were compared using Student’s *t*-test after confirming data were normally distributed (Kolmogorov–Smirnov test). A value of *p* < 0.05 was considered statistically significant.

## Results

Eighteen patients (2 females, age 72 ± 9 years; range 61–88 years) were treated with aortic aneurysm sac embolization at the same time as EVAR. Patient demographic and baseline characteristics are shown in Table 1. The majority of aneurysms were infrarenal AAAs (*n* = 16), plus an aortoiliac aneurysm extending to the external iliac artery, and a thoracic aneurysm of the proximal descending thoracic aorta. Fourteen patients underwent sac embolization alone, and the ostia of aortic side branches were embolized in addition to sac embolization in an additional 4 patients. The ostia of a total of 8 side branches were targeted for embolization in the 4 patients; 4 inferior mesenteric arteries, 3 lumbar arteries in 2 patients, and 1 internal iliac artery. The aneurysms were large, and the residual flow volume was considered large enough and with morphology able to accommodate safe delivery of the embolization devices and potentially benefit from sac embolization. A variety of aortic endografts were used, and graft selection was based on interventionalist preference for individual cases (Table 1).

**Table 1:** Baseline and procedural data

	Value (*n* = 18)		Range
Demographics			
Male sex	16	88.9	
Age, years	72	± 9	61–88
Aneurysm location			
Infrarenal	16	88.9	
Aortoiliac	1	5.6	
Thoracic	1	5.6	
Aneurysm measurements			
Diameter, mm (inner wall to inner wall)	62	± 11	53–91
Aneurysm volume, mL	195	± 117	80–444
Perfused aneurysm volume, mL	97	± 60	30–244
Residual flow lumen volume, mL^a^	63	± 49	5–200
Aortic endograft^b^			
Lombard Altura	10	55.6	
Lombard Altura fenestrated	1	5.6	
Lombard Altura w/renal chimney	1	5.6	
GORE EXCLUDER Conformable	3	16.7	
GORE TAG Conformable^c^	1	5.6	
Medtronic Endurant	1	5.6	
Endologix AFX2	1	5.6	
Shape memory polymer devices			
Number	24	± 12	5–45
Volume of embolic material, mL^d^	30	± 15	6.25–56.25
Embolic material/residual flow lumen volume^e^	108	± 119	10–433

Continuous data are presented as mean ± standard deviation. Categorical data are presented as number (% of total)

^a^Calculated by subtraction of the endograft volume (from endograft dimensions) from the perfused aneurysm volume

^b^Endograft manufacturers: Lombard Medical, Didcot, Oxfordshire, UK; W. L. Gore & Associates, Flagstaff, Ariz.; Medtronic, Minneapolis, Minn.; Endologix, Santa Rosa, CA, USA

^c^Used to treat a single patient with a thoracic aneurysm

^d^Based on the maximum expanded volume of 1.25 mL per device

^e^Ratio of the volume of shape memory polymer embolic material implanted over the calculated residual flow lumen outside of the endograft, based on the maximum expanded volume of 1.25 mL per shape memory polymer device

Technical success was achieved in all cases, with a mean of 24 ± 12 devices implanted per patient (Table 1). This included the devices positioned at the ostia of side branches to encourage thrombosis, where a mean of 3 ± 2 (range 1–6) devices were used in this manner. The mean ratio of implanted shape memory polymer volume to the calculated residual flow volume was 108% ± 119% (range 10–433%), based on the fully expanded device volume of 1.25 mL per device. The overall sac filling rate of the cohort was 50.3% (a total calculated residual flow volume of 1,063 mL and a total of 428 devices, equivalent to 535 mL of expanded porous material).

**Fig. 4:**
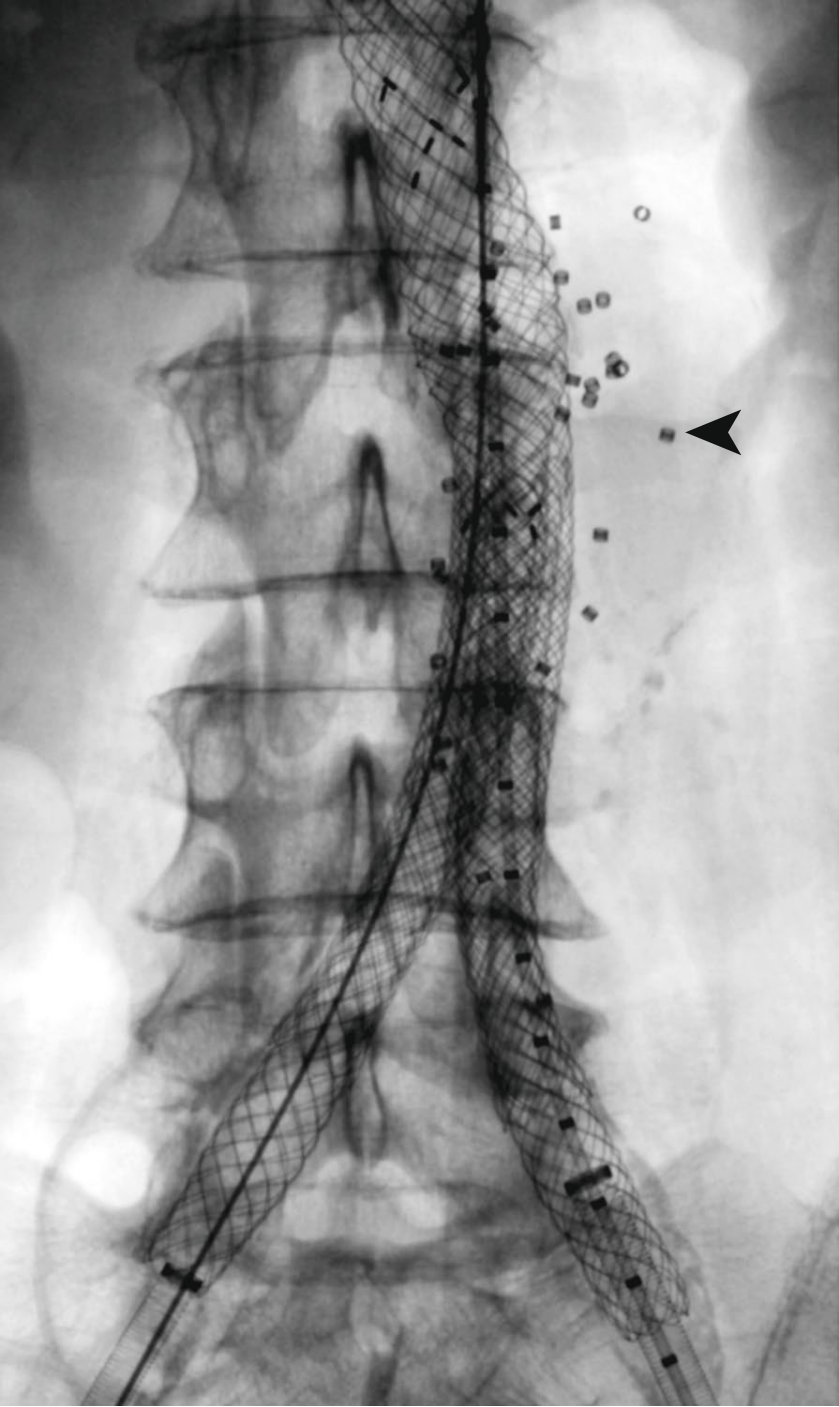
Completion fluoroscopic image post aortic endograft (Lombard Altura) deployment and implantation of 31 shape memory polymer devices, with a total expanded embolic material volume of 38.75 mL, based on 1.25 mL per device. The small proximal radiopaque markers indicate the positioning of the devices throughout the aneurysm sac behind the endograft (black arrow indicates a single device/marker)

Although the extent of sac filling was not a criterion for technical success, it was intended to fill the sac with embolic material as much as possible. However, in some cases, it was not possible to implant the intended number of devices (to fill the sac as much as possible) due to difficulties in positioning the delivery catheter at different locations in the aneurysm sac. In some patients, the number of devices implanted was larger than necessary to fill the sac based on the fully expanded device volume, which was likely due to the fast filling of the aneurysm sac before the shape memory polymer could fully expand. A representative example of fluoroscopic imaging immediately after sac embolization illustrating the distribution of the shape memory polymer devices (based on their radiopaque proximal markers) throughout an infrarenal AAA sac is shown in Fig. 4.

Aneurysm sac embolization increased procedure and fluoroscopy times by a mean of 21 ± 11 (range 10–45) min and 6 ± 4 (range, 2–15) min, respectively. The maximum additional procedure time of 45 min was while treating one of the first three patients, due to learning curve, and difficulty in advancing the aneurysm access sheath along the already deployed bifurcated aortic endograft due to atherosclerotic-related high friction.

There was no additional administration of contrast media during aneurysm sac embolization as no further angiographic imaging was necessary.

**Fig. 5:**
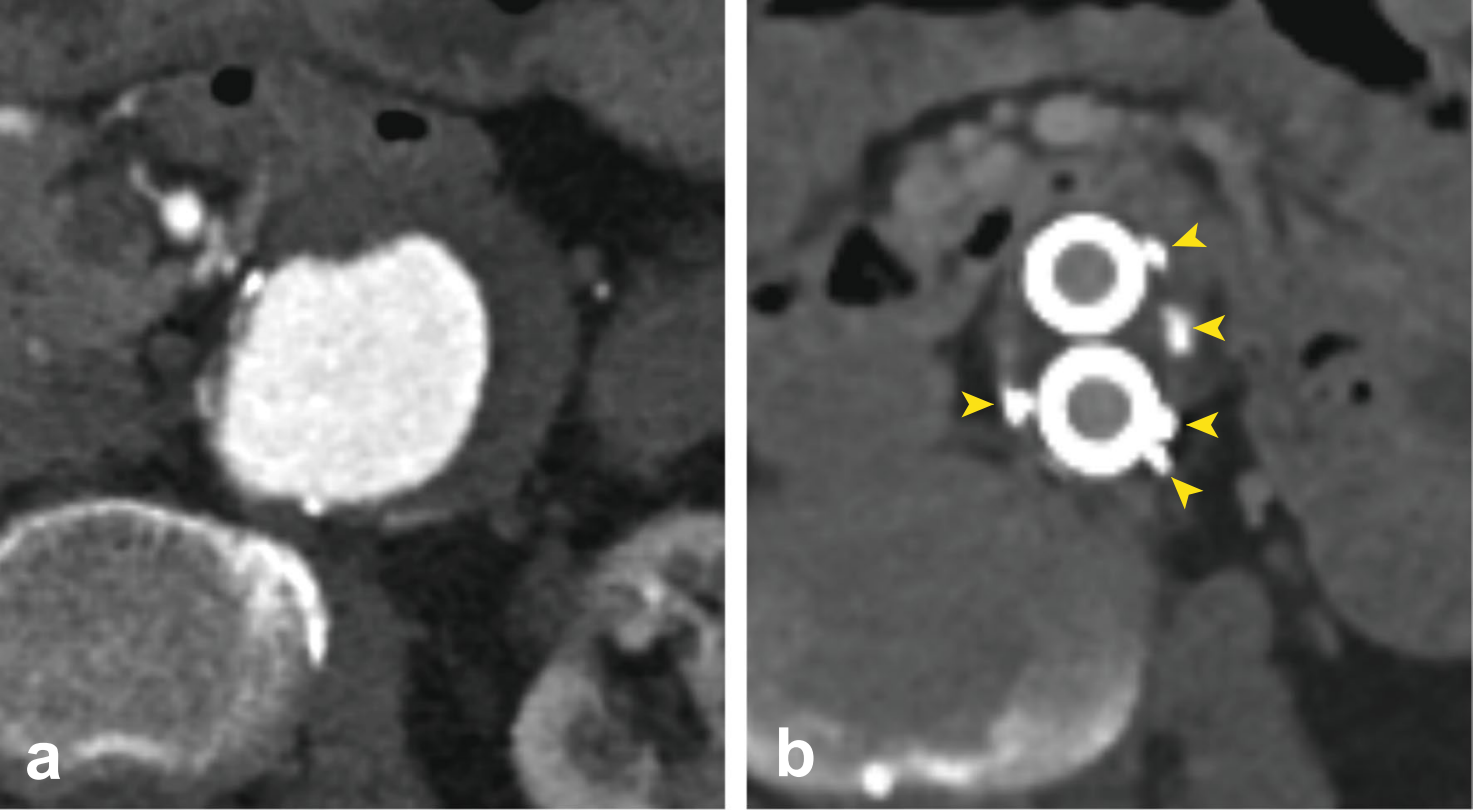
**a** Preprocedure computed tomography angiography (CTA) of a 54-mm diameter, 104-mL infrarenal AAA sac. **b** Follow-up CTA at 12 months, illustrating a reduction in aneurysm volume of 48 mL. The sac was treated with 31 shape memory polymer devices, totaling 38.75 mL of embolic material, which represented 117% of the calculated residual flow lumen volume outside of the endograft (Lombard Altura) based on preprocedural CTA analysis. Yellow arrows indicate the markers of the shape memory polymer devices

All aneurysm sacs in the 16 patients with at least 3-month imaging data decreased in volume over a mean of 11 ± 7 months (Table 2). The mean change in aneurysm volume of -30 ± 21 mL was a significant change in volume from baseline (*p* < 0.001). In the patient group with 3- to 6-month follow-up (*n* = 8), the mean change in aneurysm volume of -27 ± 20 mL was a significant change in volume from baseline (*p* = 0.018). In the patient group with 12- to 24-month follow-up (*n* = 8), the mean change in aneurysm volume of -31 ± 20 mL was a significant change in volume from baseline (*p* = 0.019). The patient group with 12- to 24-month follow-up exhibited sac regression at the 6-month mark and continued to regress through longer-term follow-up. These preliminary data indicate that aneurysm sac shrinkage occurs in all cases during the first 6 months following aneurysm sac embolization with this novel shape memory polymer embolic device. The baseline and follow-up CTAs of a patient with a reduction in aneurysm volume of 48 mL at 12 months are shown in Fig. 5.

**Table 2:** Follow-up aneurysm sac volume data based on computed tomography angiography

	Value		*p*-value^b^	Range
Follow-up, overall (*n* = 16)				
Term, months	11	± 7		3–24
Aneurysm volume, mL	194	± 134		50–442
Change in volume, mL	-30	± 21	0.0006	-2–67
% change in volume^a^	-30	± 31		-0.5 to -87
Follow-up, 3–6 months (*n* = 8)				
Aneurysm volume, mL	179	± 136		56–442
Change in volume, mL	-27	± 20	0.0181	-2 to -64
% change in volume^a^	-28	± 29		-0.5 to -87
Follow-up, 12–24 months (*n* = 8)				
Aneurysm volume, mL	195	± 125		50–404
Change in volume, mL	-31	± 21	0.0186	-8 to 67
% change in volume^a^	-30	± 31		-2 to -80

The continuous data are presented as mean ± standard deviation

^a^Mean percent change in volume from baseline values

^b^Comparing baseline to follow-up

Two-type IA endoleaks were identified by CEUS and CTA in 2 patients at 7-day follow-up but were completely resolved at 3 months without intervention. Eight type 3 endoleaks were observed in 8 patients at 7-day follow-up CEUS/CTA. In the cohort with 3–6 months follow-up, 2 endoleaks had completely resolved. The other 2 endoleaks persisted with reduced intensity and three-dimensional size. In the cohort with ≥ 12-month follow-up, 1 endoleak had completely resolved. The other 3 endoleaks were still present with reduced intensity and three-dimensional size. Three of the 4 patients with focused ostial embolization had no endoleak, and 1 patient exhibited a lumbar artery endoleak that had not been targeted for ostium embolization. Importantly, all patients that had endoleaks also had sac volume reduction, including the patients with persistent type 2 endoleaks.

There was no dislodgement of the devices from the sac during or after the procedure. There was also no off-target embolization from migration of the devices. No access site complications were observed. There was no morbidity or mortality related to aneurysm sac embolization throughout the follow-up to date. There was also no aneurysm-related morbidity or mortality in the same time frame. There were no clinical or laboratory test-based signs of inflammation; body temperature, white blood cell count, and blood sedimentation rate were within normal limits.

## Discussion

This retrospective study evaluated the performance of a newly available polymer embolization device for aortic aneurysm sac embolization at the same time as EVAR. There are numerous reports of aortic aneurysm sac embolization at the same time as EVAR with widely available embolic agents, including randomized controlled trials of the use of coils and fibrin glue *versus* no sac embolization [27, 32]. However, the use of radiopaque devices for aneurysm sac embolization hinders the ability to identify endoleaks during follow-up. Furthermore, the use of liquid or flowing embolics may result in off-target embolization and resulting complications (*e.g.*, spinal cord ischemia).

The properties of the shape memory polymer-based devices evaluated in this study are attractive for aortic sac embolization. The expanded, porous shape memory polymer material has been shown to support aneurysm sac regression [38]; the material has also been shown to support the growth of cellular, collagenous connective tissue throughout its structure without indication of a chronic inflammatory response [37]; and the majority of the device is radiolucent. Furthermore, the shape memory polymer devices self-expand to a prescribed, and therefore, predictable volume.

The instructions for use of the device also indicate the polymer bioabsorbs over time. Although each shape memory polymer device has an expanded volume of ~ 1.25 mL, it is important to recognize that the expanded porous structure of the device does not displace that volume of blood. In addition, the 1.25-mL volume comprises very little volume of polymer material, rather that the expanded, porous polymer structure serves as a scaffold supporting initial thrombus formation throughout its structure, and then followed by the formation of collagenous connective tissue intra-device.

In our experience, the deployment of shape memory polymer devices into aortic aneurysm sacs with a transfemoral parallel wire technique was relatively straightforward after a short learning curve. The devices are pushable, and the time required for device delivery did not extend the procedure or fluoroscopy time more than is reasonable, and the time is likely to decrease with increasing experience. Three of the cases were performed with the IMPEDE-FX RapidFill device after it became available, in which 5 IMPEDE-FX Embolization Plugs are loaded in an introducer and implanted at the same time. We decided to place devices at the ostia of branch vessels to encourage thrombosis if a vessel diameter was > 3 mm and patent, which is a widely acknowledged risk factor for type 2 endoleak development. The mean ratio of implanted shape memory polymer volume to the calculated residual flow volume was 108% per patient.

We acknowledge that the aortic endograft volume, and therefore the residual flow volume, may have changed on aortic endograft deployment, and therefore, this ratio is an approximation. Our intention was to fill the sac as much as possible. The shape memory polymer devices used in this study take > 5 min to expand to their predefined volume, and the expanded device has a low radial force. Consequently, we did not experience any adverse effects with filling the sacs more than 100%, based on the fully expanded volume of the device and the calculated residual flow volume. Furthermore, we did not observe any stent graft distortion.

The results of this study in terms of reduction in aneurysm sac size were encouraging, based on comparison with those reported by Piazza et al. [27] and Fabre et al. [32]. We reported our sac size data based on volume rather than diameter, since we believe volume is a more comprehensive means of measuring aneurysm sac expansion or regression. It is noteworthy that patients in our study were exhibiting a large amount of sac regression as early as 3–6 months postprocedure. In randomized controlled trials comparing with and without sac embolization, Piazza et al. and Fabre et al. reported a difference in volume-based sac regression between the two groups at 6 months and 12 months, respectively [27, 32]. Although preliminary data is in a retrospective study, our patients appear to be exhibiting larger changes in volume in the majority of patients and are continuing to regress over time. We believe this is a consequence of filling the sacs with embolic material, which is in turn enabled by the unique properties of the shape memory polymer.

The type 1 endoleaks observed in our patient set were clearly originating from the proximal sealing zone, were very small, did not warrant intervention, and resolved within 3 months. The type 2 endoleaks observed in our patients were also not significant enough to indicate intervention to date. Although all patients with endoleaks also exhibited a reduction in sac size, standard-of-care vigilance is ongoing. Piazza et al. demonstrated that outcomes may be dependent on the volume of embolic material in the sac [26, 27]. The small size of our study did not enable us to draw any conclusions on this topic; however, an increase in the amount of shape memory polymer placed in the sac may result in a decrease in the rate of endoleaks. It is noteworthy that the endoleaks were clearly visible in the presence of the shape memory polymer devices, *i.e.*, there was no artifact interference. The shape memory polymer is radiolucent but is echogenic, and Fig. 2 shows the appearance of the shape memory polymer under ultrasound.

No safety concerns arose during the cases or follow-up to date. The potential for post implantation syndrome was a concern; however, no clinical or laboratory signs of inflammation occurred. There was no migration of the devices, and the treatment of an aortoiliac aneurysm without issues suggests the shape memory polymer devices may be used for such cases if there is sufficient sealing of the iliac aneurysm with the endograft limbs to prevent caudal migration. In a total of 201 patients, Piazza et al. and Fabre et al. reported very low compilation rates and no differences in complication rates between arms in randomized controlled trials comparing the outcomes of EVAR with and without sac embolization [27, 32].

The shape memory polymer device is priced in line with other similar peripheral vessel embolization devices in Germany when used for vessel embolization. Cost analyses of sac embolization should include consideration of all aspects of postoperative care, including reinterventions, and this retrospective feasibility study is not positioned to perform such analyses. However, when comparing with and without sac embolization with coils in a randomized controlled trial, Piazza et al. noted the additional cost of sac embolization more than the offset cost of higher number of reinterventions in the arm without sac embolization [27]. Fabre et al. also noted the cost of sac embolization should balance against the cost of follow-up and secondary interventions associated with aneurysm enlargement [32].

Our initial experience with shape memory polymer embolization devices serves as a foundation for further investigation into the use of these shape memory polymer devices for active aortic aneurysm sac management as sac regression was observed in all patients after three months, even in the presence of endoleaks. Optimization of patient selection will be essential to determine which patients may benefit from active sac embolization to promote sac regression and prevent type 2 endoleaks or minimize the consequences of type 2 endoleaks. A clear requirement is sufficient non-thrombosed aneurysmal lumen for the placement of the embolic devices. Independent risk factors for type 2 endoleak are the presence of a patent inferior mesenteric artery diameter ≥ 3 mm, patent lumbar, sacral or renal accessory arteries ≥ 2 mm, and aortoiliac aneurysm extension [39]. Based on these criteria, ~ 50% of patients referred for EVAR would be potential candidates for active aortic aneurysm sac embolization [21]. Moreover, indication for active sac management with embolization should be considered in terms of patient age, comorbidities, and aneurysm volume [39].

This retrospective feasibility study has obvious limitations, including lack of a control arm/comparison with standard EVAR. The retrospective nature of the study means inclusion/exclusion criteria were not predefined. The imaging data were analyzed retrospectively without core lab adjudication, and the study is small with limited follow-up of some patients to date. This study represents initial experience and with a variety of aortic endografts and different amounts of embolic material.

In conclusion, the use of shape memory polymer devices for the active treatment of aortic aneurysm sacs appears feasible based on this small case series. Technical success of the delivery of shape memory polymer devices into the residual flow lumen behind aortic endografts was achieved in all cases. A wide range aortic endografts were used. No safety concerns arose to date. The volume-based sac regression observed over the medium term supports further investigation into the potential of using this embolic material in active aortic aneurysm sac management. Prospective studies are needed to evaluate the effectiveness of active aneurysm sac management with these novel devices in specific patient populations.

## Supplementary Information

**Additional file 1: Video 1.** Fluoroscopy IMPEDE deployment.

## Data Availability

All datasets on which the conclusions of the manuscript rely are presented in the main paper.

## Abbreviations

AAA: Abdominal aortic aneurysm
CEUS: Contrast-enhanced ultrasound
CTA: Computed tomography angiography
EVAR: Endovascular aneurysm repair
